# Supplementary material for: Effects of Ionic Strength on Lateral Particle Migration in Shear-Thinning Xanthan Gum Solutions
Source: Micromachines (Basel). 2019 Aug 15;10(8):535. doi: 10.3390/mi10080535 (PMC6723194; doi:10.3390/mi10080535)
Supplement: Supplementary file 1 [file micromachines-10-00535-s001.pdf]

micromachines

Supplementary Information

Effects of Ionic Strength on Lateral Particle Migration in Shear-Thinning Xanthan Gum Solutions

Mira Cho, Sun Ok Hong, Seung Hak Lee, Kyu Hyun and Ju Min Kim

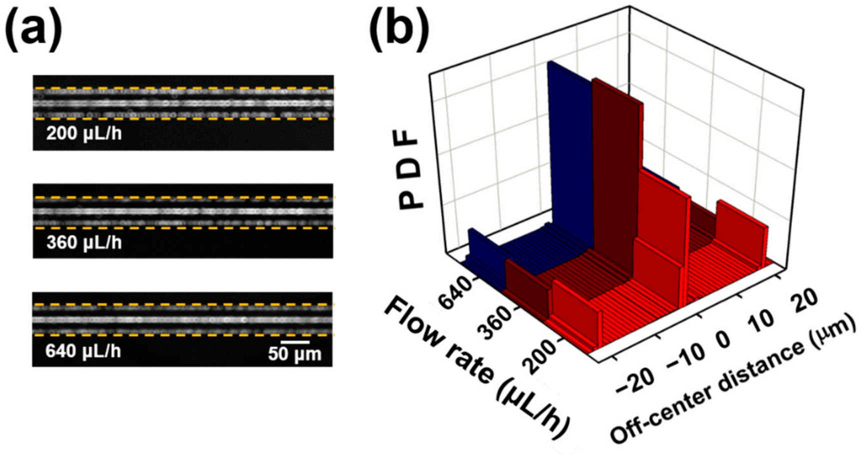

Figure S1. Particle distributions according to flow rates in 6.4 wt % poly(vinyl pyrrolidone) (PVP) aqueous solution: standard deviation images (left column) and particle distribution function (PDF). The standard deviation images were obtained by stacking 2000 successive time-lapse images with the standard deviation option in Image J software.

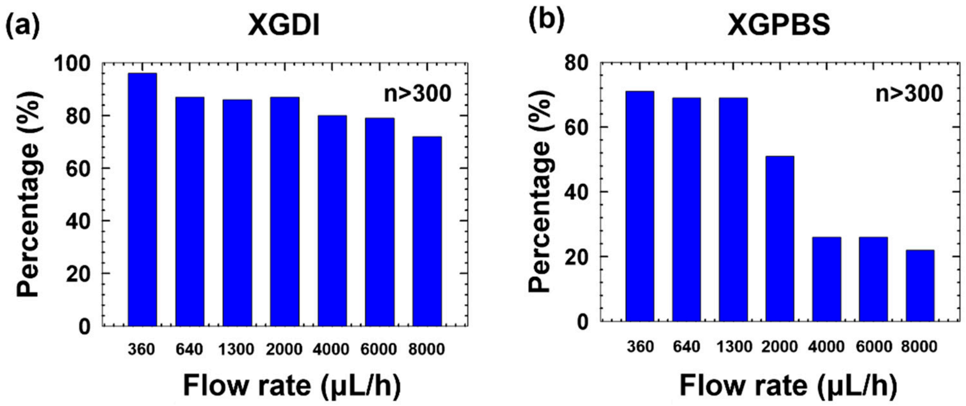

Figure S2. (**a**) Probability of localization of particles along the centerline (approximately ±3 μm from the centerline) in xanthan gum (XG) solution in deionized water (XGDI). (**b**) Probability of particle localization along the corners in XG solution in phosphate buffered saline (PBS) solution (XGPBS). More than 300 particles in high-speed movies were examined to determine the probabilities at each flow rate.

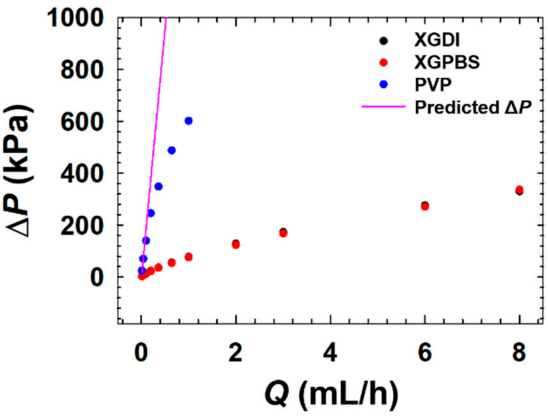

Figure S3. Measured pressure drops between the channel inlet and outlet in the xanthan gum (XG) solutions (XGDI and XGPBS) and poly(vinyl pyrrolidone) (PVP) solution (PVP). The pink straight line denotes the theoretical prediction in the Newtonian fluid (refer to the Experimental section in the main text for the details of the measurement methods and the theoretical prediction in Newtonian fluid). The theoretical prediction is in good agreement with the experimental data for the PVP solution at low flow rates, but deviates from the experimental data at high flow rates (≥ 0.4 mL·h^{-1}), which can be attributed to swelling of the flexible poly(dimethyl siloxane) channel at high flow rates.

© 2019 by the authors. Submitted for possible open access publication under the terms and conditions of the Creative Commons Attribution (CC BY) license (http://creativecommons.org/licenses/by/4.0/).
